# The JNK Signaling Pathway in Renal Fibrosis

**DOI:** 10.3389/fphys.2017.00829

**Published:** 2017-10-24

**Authors:** Keren Grynberg, Frank Y. Ma, David J. Nikolic-Paterson

**Affiliations:** Department of Nephrology, Monash Medical Centre, Monash University Centre for Inflammatory Diseases, Monash Health, Clayton, VIC, Australia

**Keywords:** apoptosis, ASK1, kidney disease, kidney inflammation, p38 MAPK, SMAD3

## Abstract

Fibrosis of the glomerular and tubulointerstitial compartments is a common feature of chronic kidney disease leading to end-stage renal failure. This fibrotic process involves a number of pathologic mechanisms, including cell death and inflammation. This review focuses on the role of the c-Jun amino terminal kinase (JNK) signaling pathway in the development of renal fibrosis. The JNK pathway is activated in response to various cellular stresses and plays an important role in cell death and inflammation. Activation of JNK signaling is a common feature in most forms of human kidney injury, evident in both intrinsic glomerular and tubular cells as well as in infiltrating leukocytes. Similar patterns of JNK activation are evident in animal models of acute and chronic renal injury. Administration of JNK inhibitors can protect against acute kidney injury and suppress the development of glomerulosclerosis and tubulointerstitial fibrosis. In particular, JNK activation in tubular epithelial cells may be a pivotal mechanism in determining the outcome of both acute kidney injury and progression of chronic kidney disease. JNK signaling promotes tubular epithelial cell production of pro-inflammatory and pro-fibrotic molecules as well as tubular cell de-differentiation toward a mesenchymal phenotype. However, the role of JNK within renal fibroblasts is less well-characterized. The JNK pathway interacts with other pro-fibrotic pathways, most notable with the TGF-β/SMAD pathway. JNK activation can augment TGF-β gene transcription, induce expression of enzymes that activate the latent form of TGF-β, and JNK directly phosphorylates SMAD3 to enhance transcription of pro-fibrotic molecules. In conclusion, JNK signaling plays an integral role in several key mechanisms operating in renal fibrosis. Targeting of JNK enzymes has therapeutic potential for the treatment of fibrotic kidney diseases.

## Introduction

Kidney disease can be classified as acute or chronic and can affect the glomerular, tubular, and interstitial compartments of the kidney. Acute kidney injury can be directed at the glomerulus (glomerulonephritis), tubules (acute tubular necrosis), or interstitium (acute interstitial nephritis). Precipitants of acute kidney injury are varied but include immunological, hypoxic, and toxin mediated causes. A wide range of insults including immunity, hypertension, diabetes, and prior acute injury can induce progressive chronic disease which is hallmarked by inflammation and fibrosis leading to end-stage kidney disease.

Renal fibrosis involves a loss of renal parenchyma and an excessive deposition of extra-cellular matrix. This is evident in all progressive forms of chronic kidney disease irrespective of the initial injury. Glomerulosclerosis and, in particular, interstitial fibrosis correlates with loss of renal function and is considered a common pathway leading to end stage renal disease (Mackensen-Haen et al., 1981; Nath, 1992). A wide range of factors can promote renal fibrosis of which pathologic cell death and chronic inflammation are considered to be major mechanisms underpinning this destructive process (Meng et al., 2014). Tubular epithelial cells can become active drivers of the inflammatory and fibrotic processes following either direct tubular damage or tubular damage secondary to glomerular injury (Meng et al., 2014). A number of signaling pathways have been implicated in the fibrotic process (e.g., TGF-β/SMAD, Wnt/β-catenin, Jagged/Notch, EGF-R, and JAK/STAT). This review focuses on the role of the c-Jun amino terminal kinase (JNK) in renal fibrosis.

## Overview of the JNK pathway

**Figure 1:**
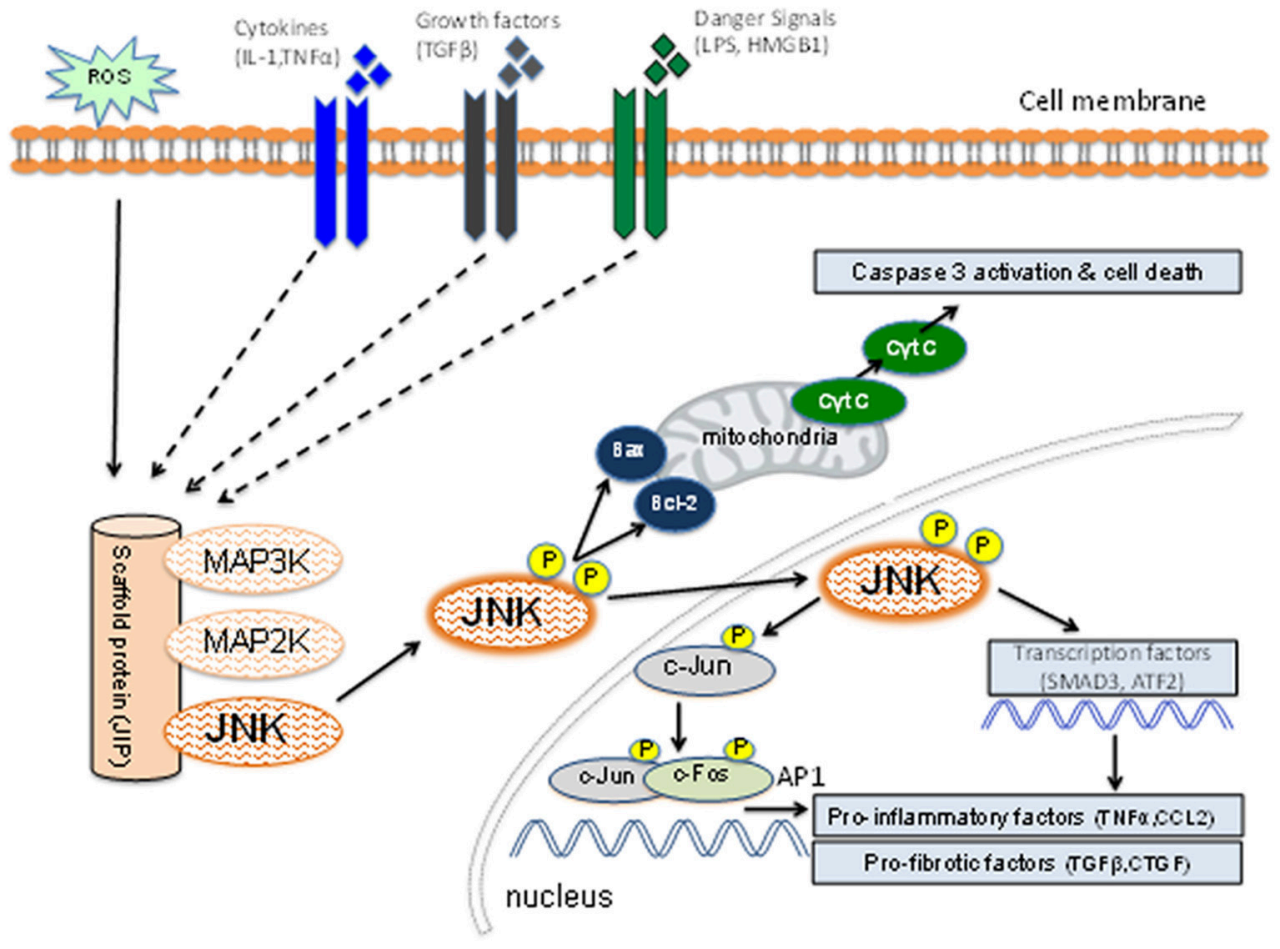
Schematic diagram of the JNK signaling pathway. Inflammatory cytokines, danger-associated molecules pattern ligands (alarmins), and pro-fibrotic growth factors can induce activation of members of the mitogen-activated protein kinase kinase kinase (MAP3K) family. Other factors such as reactive oxygen species (ROS) and osmotic stress can also lead to MAP3K activation. Individual members of the MAP3K and MAP2K families plus JNK are held in close proximity by the scaffold protein (JNK-interacting protein-1) which facilitates a rapid series of phosphorylation reactions leading to phosphorylation of the activation site of JNK. The activated JNK then dissociates from this complex and can promote mitochondria-dependent apoptosis through B cell lymphoma (Bcl-2) and Bcl-2 associated x-protein (Bax). Active JNK can promote transcription of genes involved in the inflammatory and fibrotic responses through phosphorylation of c-Jun [to promote activator protein-1 (AP-1) function], SMAD3 and ATF2. JNK activation can promote cell death, inflammation and fibrosis.

JNK is one of three well-characterized mitogen-activated protein kinase (MAPK) pathways which transduce extra-cellular signals to control processes such as cell proliferation, differentiation, migration, and apoptosis (Bode and Dong, 2007). Each of these MAPK pathways are activated via a cascade of phosphorylation reactions. In the case of JNK, upstream MAP3K family members phosphorylate and activate MAP2K enzymes (MKK4 and MKK7), which, in turn, phosphorylate and activate JNK (Figure 1). Rapid activation of the pathway is facilitated by scaffold proteins, such as JNK interacting protein 1 (JIP1) (Avruch, 2007). On the other hand, inactivation of the JNK pathway occurs by dephosphorylation performed by a group of dual specificity phosphatases (Patterson et al., 2009).

Upon activation, JNK can phosphorylate serine and threonine residues in specific protein substrates. JNK derives its name from its ability to phosphorylate serine residues at positions 63 and 73 in the amino-terminal domain of the c-Jun proto-oncogene (Bode and Dong, 2007; Wagner and Nebreda, 2009). There are three genes in the JNK family (*Jnk1/Mapk8, Jnk2/Mapk9*, and *Jnk3/Mapk10*). JNK1 and JNK2 enzymes are expressed in most cells of the body, including the kidney, whereas expression of JNK3 is limited to the brain, heart, and testis (Gupta et al., 1996; Kumar et al., 2015). Deletion of both *Jnk1* and *Jnk2* genes causes fetal lethality, whereas deletion of either *Jnk1* or *Jnk2* results in viable and healthy mice (Wagner and Nebreda, 2009). Alternative splicing of mRNA from these three genes gives rise to at least 10 different JNK enzyme isoforms ranging between 46 and 55 kDa (Gupta et al., 1996). The JNK pathway is broadly expressed but context specific, indicating that specific stimuli can activate distinct cellular pathways and responses (Davis, 2000).

An important aspect of the JNK pathway is that it can be activated in response to a range of stimuli that have been implicated in acute and chronic kidney injury, including: pro-inflammatory cytokines, danger-associated molecules pattern ligands (alarmins), oxidative stress, pro-fibrotic factors, and nephrotoxins (Figure 1). Thus, JNK signaling may contribute to renal fibrosis through induction of apoptosis and inflammation as well as a direct contribution to fibrosis itself.

### JNK in apoptosis

JNK is best known for its role in the induction of cell apoptosis (Davis, 2000). JNK is directly involved in the mitochondrial (or intrinsic) pathway of apoptosis (Figure 1). Activation of JNK by oxidative stress causes mitochondrial release of cytochrome c into the cytoplasm leading to caspase activation and apoptosis (Tournier et al., 2000). This is thought to operate via B cell lymphoma (Bcl-2) and Bcl-2 like 1 gene (Bcl-xs) oncoproteins on the surface of mitochondria (Maundrell et al., 1997). However, JNK signaling is not always pro-apoptotic and can be context specific. For example, in fibroblasts JNK can act to suppress TNF-stimulated apoptosis, but JNK can also potentiate TNF-stimulated cell death via increased production of reactive oxygen species (Ventura et al., 2004). Of note, JNK activation can also promote cell survival through the induction of autophagy. Under conditions of cell starvation, JNK1 but not JNK2, can phosphorylate Bcl-2 causing disruption of the Bcl-2/beclin-1 complex and activation of the autophagy response (Wei et al., 2008).

### JNK in inflammation

The JNK pathway can promote an inflammatory response in both leukocytes and non-leukocytes. Although the nature of the stimuli inducing JNK activation may vary depending upon the cell type and nature of tissue injury, a primary mechanism through which the JNK pathway promotes inflammation is the transcription factor, AP-1 (Figure 1). JNK can phosphorylate c-Jun which enables dimerization with c-Fos to make AP-1 which transcribes a wide range of genes that orchestrate the inflammatory response, including cytokines (e.g., TNF-α), chemokines (e.g., CCL2), and leukocyte adhesion molecules (e.g., VCAM-1/CD106) (Ip and Davis, 1998). Thus, activation of JNK in endothelial cells can facilitate adhesion and transmigration of leukocytes via up-regulation of chemokines and adhesion molecules, while JNK activation in epithelial cells (e.g., in the injured kidney, lung, or liver) can recruit and activate leukocyte populations via chemokine and cytokine production. A second phosphorylation target of JNK is activating transcription factor 2 (ATF-2) which also transcribes genes that contribute to the inflammatory response (Yu et al., 2014). In addition, JNK signaling also plays a role in activation of Th1 and Th2 subsets of T cells (Davis, 2000).

Nuclear factor-kappaB (NF-kB) is a major factor that promotes transcription of genes involved in the inflammatory and anti-apoptotic responses (Workman and Habelhah, 2013). Many of the same stimuli (e.g., TNF-α, IL-1, LPS, oxidative stress) can induce both JNK/AP-1 and NF-kB activation which drives inflammation (Workman and Habelhah, 2013). In addition, JNK can directly activate NF-kB by promoting IkBα degradation (Ruan et al., 2015). Thus, while JNK/AP1 and NF-kB can coordinate the inflammatory response, NF-kB acts to limit JNK-dependent cell death (Workman and Habelhah, 2013; Ruan et al., 2015).

Thus, activation of the JNK pathway has the potential to promote renal fibrosis through its pro-apoptotic and pro-inflammatory actions. In addition, as discussed below, there is now evidence that JNK signaling can act directly to potentiate the fibrotic response.

### Inhibition of the JNK pathway

A standard approach to defining the function of a signaling pathway is through genetic and/or pharmacologic inhibition. In the case of JNK signaling, deletion of one or more of *Jnk1-3* genes has been highly informative both in terms of individual gene function and for identifying redundancy in the signaling pathway (Wagner and Nebreda, 2009). Two main approaches have been used for pharmacologic inhibition of JNK activity. A series of small molecule inhibitors such as SP600125 and CC-401 have been developed which inhibit JNK1-3 with IC_50_ values of 25–90 mM (Bennett et al., 2001; Ma et al., 2007a), while CC-930 inhibits JNK2/3 (6–7 nM) more potently than JNK1 (61 nM) (Plantevin Krenitsky et al., 2012). A second approach has been to block the JNK-interacting protein 1 (JIP1) scaffold protein from binding JNK enzymes. This utilizes a 20 amino acid peptide from the JIP1 protein to compete for JNK binding. Conjugated of the JIP1 peptide with a 10 amino acid HIV–TAT transporter sequence provides a cell-permeable molecule that can inhibit all JNK enzymes (Vivès et al., 1997). These strategies have been instrumental in determining the role of JNK in kidney injury and fibrosis.

## JNK signaling in glomerular disease

Two main approaches have been used to detect activation of the JNK pathway in tissues. First, tissue lysates can be examined for phosphorylation of the activate site of the JNK enzyme (JNK^Tyr183/185^) by Western blotting or JNK activity can be directly measured in a kinase assay. Second, immunohistochemistry staining for phospho-JNK^Tyr183/185^ can identify and localize cell types in which JNK signaling occurs. An indirect method to assess JNK signaling in tissue lysates or sections is to measure c-Jun^Ser63^ phosphorylation (referred to as p-c-Jun) since JNK is the only kinase known to phosphorylate c-Jun in this position.

Immunohistochemistry staining has identified JNK activation in collecting duct and parietal epithelial cells in normal mouse, rat and human kidney (Flanc et al., 2007; Ma et al., 2007a; Kanellis et al., 2010). While p-c-Jun staining can be seen in a small number of parietal epithelial cells in normal kidney, this is not apparent in collecting ducts which lack c-Jun expression under normal conditions (De Borst et al., 2007; Ma et al., 2007a; Kanellis et al., 2010).

### JNK activation in glomerular disease

Immunohistochemistry staining for p-JNK and p-c-Jun in renal biopsies have identified marked JNK activation in most types of glomerulonephritis as well as in diabetic nephropathy (De Borst et al., 2007; de Borst et al., 2009). Numerous glomerular cells exhibit JNK activation across all diseases and this correlates with the degree of macrophage infiltration and glomerulosclerosis, but not with proteinuria or renal function (De Borst et al., 2007; de Borst et al., 2009).

JNK activation has also been identified in a diverse range of animal models of glomerular disease, including; crescentic glomerulonephritis, diabetic nephropathy, minimal change disease, Alport's syndrome, and salt-sensitive hypertension (Nishiyama et al., 2004; Park and Jeong, 2004; Flanc et al., 2007; Lim et al., 2011; Nakagawa et al., 2016). In addition, increased JNK activation has been identified in the aging kidney (Kim et al., 2002).

### JNK blockade in experimental glomerular disease

**Figure 2:**
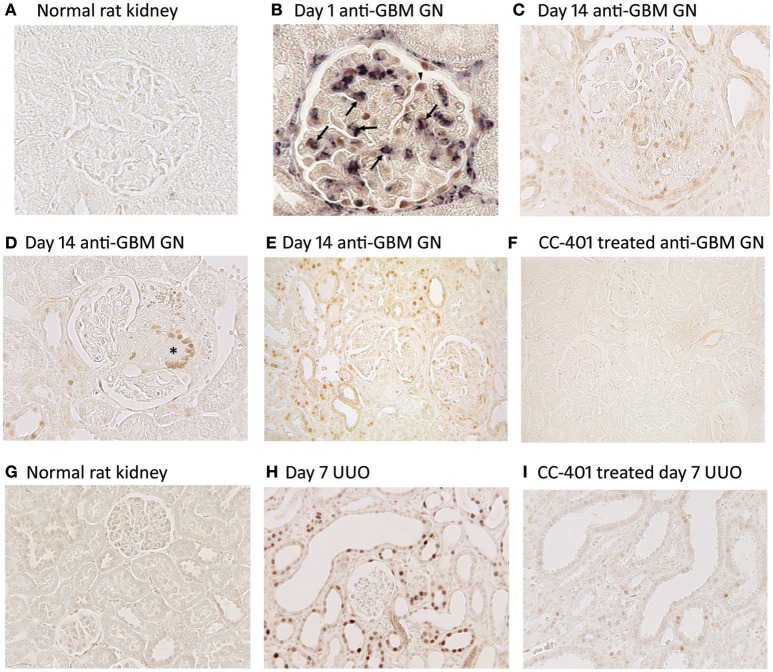
JNK activation in kidney injury. Detection of JNK activation by immunohistochemistry staining for c-Jun phosphorylated at Ser63 (p-c-Jun). **(A–F)** JNK activation in rat anti-glomerular basement membrane (GBM) glomerulonephritis (GN). **(A)** Normal rat glomeruli lack p-c-Jun staining. **(B)** Two color immunostaining for p-c-Jun (brown nuclei) and ED1+ macrophages (purple/blue) in a glomerulus on day 1 of anti-GBM disease. Arrows indicate cells stained for both ED1 and p-c-Jun, demonstrating JNK signaling in macrophages. Arrowhead shows p-c-Jun immunostaining in a podocyte-like cell. Parietal epithelial cells staining for p-c-Jun is also evident. **(C)** Glomerulus on day 24 of anti-GBM disease shows nuclear p-c-Jun staining of cells in the capillary tuft and in the crescent. **(D)** Day 24 of anti-GBM disease showing p-c-Jun staining in a multinucleated giant cell in a glomerular crescent (^*^). **(E)** Lower power view of day 24 anti-GBM disease showing p-c-Jun+ cells in the glomerular tuft, in crescents, in many tubular epithelial cells and in some interstitial cells. **(F)** Treatment with the JNK inhibitor CC-401 over days 7–24 of anti-GBM disease blocks c-Jun phosphorylation. **(G–I)** JNK activation in the rat unilateral ureteric obstruction (UUO) model. **(G)** Normal rat kidney shows a lack of p-c-Jun staining in glomeruli and tubules. **(H)** Day 7 after UUO shows extensive JNK activation in both atrophic tubules and in tubules with normal morphology. Glomeruli do not show inflammation in this model and they lack p-c-Jun staining. **(I)** Treatment with the JNK inhibitor CC-401 over days 0–7 of the UUO model blocks c-Jun phosphorylation. Reproduced with permission from Flanc et al. (2007) **(A,B)**; Ma et al. (2009) **(C–F)**, and; Ma et al. (2007a) **(G–I)**.

Acute anti-glomerular basement membrane (GBM) glomerulonephritis features JNK activation in glomerular podocytes and parietal epithelial cells as well as infiltrating macrophages (Figures 2A,B). As disease progresses, JNK activation becomes evident in cells within glomerular crescents, including multi-nucleated macrophage giant cells, together with extensive JNK activation in tubular epithelial cells during secondary tubulointerstitial damage (Figures 2C–E). Administration of a JNK inhibitor (CC-401) abrogated phosphorylation of c-Jun in diseased kidneys demonstrating effective JNK blockade (Figure 2F). This resulted in significant protection from acute and progressive proteinuria. JNK blockade also protected against glomerular damage, including crescent formation and sclerosis (Flanc et al., 2007). However, this protection was independent of glomerular neutrophil and T cell infiltration. In a subsequent study, intervention with CC-401 during ongoing renal injury prevented crescent formation and halted the development of renal failure and renal fibrosis (Ma et al., 2009). While JNK blockade did not prevent macrophage recruitment into the kidney, it did suppress the M1 pro-inflammatory macrophage response (Flanc et al., 2007; Ma et al., 2009). This is consistent with the established role for macrophages in causing renal injury in this model (Han et al., 2011), and with adoptive transfer studies in which selective JNK blockade in macrophages prevented their activation within the glomerulus and thereby prevented acute glomerular injury in this model (Ikezumi et al., 2004).

In contrast to the anti-GBM model, JNK signaling does not play a clear pathologic role in diabetic nephropathy. Activation of JNK has been described in human diabetic nephropathy (De Borst et al., 2007), streptozotocin-induced diabetic nephropathy in the spontaneous hypertensive rat and in the *db/db* model of spontaneous type 2 diabetic nephropathy (Ijaz et al., 2009; Lim et al., 2011). However, blockade of JNK signaling using either a selective kinase inhibitor (CC-930) or a JIP1 peptide-based inhibitor failed to protect the kidney from mesangial expansion and caused a modest increase in the severity of podocyte damage and albuminuria (Ijaz et al., 2009; Lim et al., 2011). Why JNK blockade exacerbated podocyte damage in diabetic nephropathy is unclear since JNK blockade protects cultured podocytes against injury caused by TNF-α or by antibody plus complement (Peng et al., 2002; Ikezumi et al., 2008).

Of note, podocytes express JunD, an atypical component of the AP-1 family, and JunD confers protection against glomerlosclerosis in anti-GBM disease and subtotal nephrectomy models (Pillebout et al., 2003; Hernandez et al., 2008; Cook et al., 2011). While JNK can phosphorylate and activate JunD, it is not the only kinase which can perform this function, and this redundancy probably explains why JNK blockade is protective in anti-GBM disease whereas *JunD* gene deletion is detrimental (Flanc et al., 2007; Ma et al., 2009; Cook et al., 2011). Activation of the JNK pathway may also facilitate glomerulosclerosis through PDGF and angiotensin II induced proliferation of mesangial cells (Kawano et al., 2003; Zhang et al., 2005).

JNK activation has also been described in glomerular endothelial cells (De Borst et al., 2007). Previous studies have shown that cytokine induced *Ccl2* gene transcription in cultured human endothelial cells depends on the cooperative action of AP1 and NF-kB (Martin et al., 1997), and that uremic toxins can activate glomerular endothelial cells via JNK signaling (Shen et al., 2016). However, a direct role for endothelial cell JNK activation in regulating glomerular inflammation remains to be demonstrated.

In summary, JNK signaling can promote glomerular necrosis and fibrosis via effects upon macrophages, endothelial cells, and mesangial cells. JNK signaling can induce podocyte damage and albuminuria, while JNK activation promotes glomerular crescent formation via effects upon macrophages and possibly on parietal epithelial cells.

## JNK signaling in tubulointerstitial injury and fibrosis

**Figure 3:**
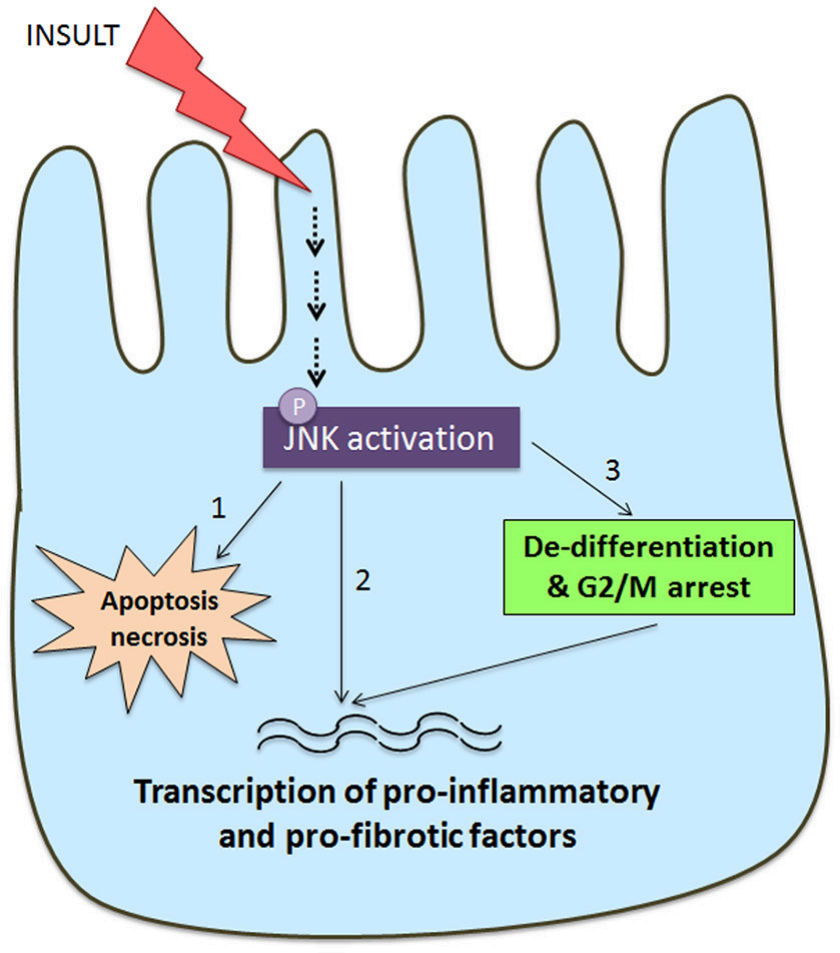
Schematic diagram of JNK signaling in the tubular epithelial cell response to damage. Many insults to the tubular epithelial cell, such as oxidative stress and nephrotoxins, as well as cytokines and growth factors can result in activation of the JNK pathway. JNK signaling in tubular epithelial cells can result in tubular cell damage and consequent tubulointerstitial damage via three pathways. (1) JNK activation can directly induce apoptosis and necrosis via mitochondrial dependent mechanisms. (2) JNK phosphorylates a number of transcription factors (e.g., c-Jun, SMAD3, ATF2) resulting in tubular production of pro-inflammatory and pro-fibrotic molecules that promote recruitment and activation of macrophages and myofibroblasts. (3) JNK activation is implicated TGF-β induced de-differentiation of tubular cells toward a partial mesenchymal phenotype with production of pro-fibrotic factors. This switch toward a pro-fibrotic mesenchymal phenotype can also be induced by JNK-dependent arrest at the G2/M phase of the cell cycle when tubular cells are undergoing a repair response.

JNK signaling in tubular epithelial cells can be seen as a key determinant in the development of tubulointerstitial injury and fibrosis based upon the common finding of tubular JNK activation in human and experimental kidney disease. This can result in cell death via apoptosis or necrosis, activation of pro-inflammatory and pro-fibrotic responses, and tubular cell de-differentiation leading to activation of a mesenchymal gene signature (Figure 3).

Prominent JNK activation in many tubular epithelial cells is a striking aspect of many forms of human glomerular disease (De Borst et al., 2007; de Borst et al., 2009). Indeed, JNK activation in tubulointerstitial cells gave a significant correlation with declining renal function, macrophage infiltration, tubular damage, and interstitial fibrosis (De Borst et al., 2007; de Borst et al., 2009). Two color immunohistochemistry identified JNK activation in damaged tubules and in areas of macrophage infiltration and fibrosis (De Borst et al., 2007), suggesting an active role for tubular JNK signaling in this process. Support for this proposal comes from studies of animal models of anti-GBM glomerulonephritis and Alport's syndrome in which prominent tubular JNK activation develops secondary to glomerular injury (Ma et al., 2009; Nakagawa et al., 2016). Indeed, commencing JNK inhibitor treatment (CC-401) in established anti-GBM disease resulted in marked protection from interstitial macrophage infiltration, tubular damage and interstitial fibrosis (Ma et al., 2009); however, this could also be, in part, due to inhibition of the primary glomerular injury.

### JNK in acute kidney injury

It is now well-recognized that episodes of acute kidney injury (AKI) are linked to subsequent development of chronic kidney disease (Zuk and Bonventre, 2016), emphasizing the importance of limiting the severity of AKI. A number of factors can induce AKI including sepsis, drug toxicity, and ischaemia. Indeed, AKI is a predictable outcome in a number of settings, including cardiac bypass surgery, kidney transplantation, and treatment with chemotherapeutic drugs such as cisplatin.

Tubular epithelial cells exhibit dramatic activation of the JNK pathway within minutes of reperfusion of the ischaemic kidney. This has been identified in post perfusion human renal transplant biopsies (Kanellis et al., 2010). The highest levels of JNK activation were seen in kidneys from deceased donors when compared with kidneys from living donors, with the degree of JNK activation correlating with ischaemic time in deceased donor allografts (Kanellis et al., 2010). Animal models recapitulate this rapid JNK activation in tubular epithelial cells following ischaemia/reperfusion (I/R) injury (Wang et al., 2007; de Borst et al., 2009; Kanellis et al., 2010). Systemic treatment with a JNK inhibitor (CC-401 or SP600125) prior to bilateral renal I/R injury protects against tubular damage and acute renal failure (Wang et al., 2007; Kanellis et al., 2010). Notably, delaying JNK inhibitor treatment until 1 h after reperfusion conferred no benefit, confirming that the early peak of JNK activation is the main pathologic event to be targeted (Kanellis et al., 2010). The postulated mechanisms for the protection seen with systemic JNK blockade are reduced tubular necrosis and apoptosis, reduced expression of pro-inflammatory molecules (e.g., TNF-α and CCL2) by tubular epithelial cells and reduced interstitial accumulation and activation of macrophages and T-cells (Wang et al., 2007; de Borst et al., 2009; Kanellis et al., 2010).

Endothelial cell activation is an important mechanism in renal I/R injury with blockade of the adhesion molecule CD54 (ICAM-1) suppressing leukocyte recruitment and renal injury (Kelly et al., 1996). Systemic delivery of a JNK inhibitor (CC-401) reduced endothelial and tubular CD54 expression and infiltration by T cells and macrophages (Kanellis et al., 2010). A direct role for endothelial JNK activation in inflammation is suggested by studies in which JNK blockade in cultured microvascular endothelial cells prevented diapedesis of CD4+ T cells across the endothelial layer (Dragoni et al., 2017). Furthermore, the direct delivery of a JIP-1 peptide-based JNK inhibitor to the renal endothelium via renal artery infusion reduced the severity of renal dysfunction in subsequent renal I/R injury, although leukocyte recruitment was not investigated (Doi et al., 2013).

Acute kidney injury induced by the nephrotoxin cisplatin is also associated with prominent tubular JNK activation, particularly in older mice which are more susceptible to developing AKI (Guan et al., 2017). Administration of a JNK inhibitor (SP600125) substantially protected against cisplatin-induced tubular damage and acute renal failure (Francescato et al., 2007). Indeed, numerous *in vitro* studies have shown that JNK signaling directly contributes to tubular epithelial cell death in response to noxious stimuli such as ATP depletion (Ma and Devarajan, 2008), reactive oxygen species (Pat et al., 2003; Arany et al., 2004), and nephrotoxic molecules (de Graauw et al., 2005). Thus, there is good evidence to conclude that JNK activation in tubular epithelial cells is a key determinant in the outcome of acute kidney injury.

### JNK in primary tubulointerstitial fibrosis

JNK activation in tubular epithelial cells is a common response to various stressors. The role of JNK signaling in renal interstitial fibrosis was first examined using unilateral ureteric obstruction (UUO). The UUO model features a rapid development of interstitial fibrosis due to a primary stretch-induced injury to tubular epithelial cells. There is dramatic and persistent JNK signaling in dilated and atrophic tubules as well as in tubules with normal morphology following ureteric obstruction (Figures 2G,H). Gene deletion of either *Jnk1* or *Jnk2* did not affect the prominent tubular JNK activation in the obstructed kidney or the development of renal fibrosis, indicating redundancy between JNK1 and JNK2 in this model (Ma et al., 2007a). However, treatment with a JNK inhibitor (CC-401) efficiently blocked JNK signaling (Figure 2I), and provided substantial protection against interstitial fibrosis in terms of myofibroblast accumulation and collagen deposition, as well as reducing mRNA levels of transforming growth factor-β1 (TGF-β1) and connective tissue growth factor (Ma et al., 2007a). Tubular apoptosis is also evident on day 7 of the UUO model and this was significantly reduced in *Jnk1* (but not *Jnk2*) deficient mice as well as with CC-401 treatment, providing the first demonstration that JNK1 has a selective role in apoptosis of kidney cells (Ma et al., 2007a).

### JNK and tubular de-differentiation

The contribution of tubular epithelial cells to the development of renal interstitial fibrosis is a topic of great interest. While tubular epithelial cells can undergo complete morphologic and functional transition into myofibroblasts in a TGF-β/JNK dependent mechanism *in vitro* (Fan et al., 1999; Mariasegaram et al., 2010), this transition is a rare event *in vivo* in human and experimental kidney disease (Ng et al., 1998; Jinde et al., 2001). Recent lineage tracing studies in mouse models of kidney disease have either found no evidence to support this mechanism (Grgic et al., 2012), or suggest that tubular epithelial cells account for less than 5% of interstitial fibroblasts (LeBleu et al., 2013). Nevertheless, studies have shown that even partial de-differentiation of tubular epithelial cells—as a result of incomplete repair following tubular cell injury—can result in expression of a mesenchymal and pro-fibrotic gene expression program (Yang et al., 2010; Lan et al., 2012; Zhu et al., 2016). Using several models of tubular injury (severe I/R injury, UUO, and aristolochic acid nephropathy), a subset of tubular epithelial cells were shown to be arrested in the G2/M phase of the cell cycle (Yang et al., 2010). *In vitro* studies identified a role for JNK in aristolochic acid-induced G2/M arrest in cultured tubular epithelial cells, and administration of a JNK inhibitor (SP600125) in a model of severe I/R injury suppressed both cell cycle arrest and development of interstitial fibrosis (Yang et al., 2010). Consistent with this finding, JNK activation is seen in tubular epithelial cells with *de novo* vimentin expression (a marker of de-differentiation) on day 14 after severe I/R injury and in human glomerular disease featuring severe tubulointerstitial damage (Lan et al., 2012). In addition, the differentiation factor Numb has been identified as promoting tubular cell G2/M arrest during interstitial fibrosis in association with activation of JNK signaling (Zhu et al., 2016). However, a study of cultured tubular epithelial cells found that JNK inhibition could suppress TGF-β1 induced PDGF-B production, but did not affect G2/M cell cycle arrest (Wu et al., 2013), questioning whether these two responses are directly linked.

Direct evidence that tubular de-differentiation drives interstitial fibrosis comes from studies using conditional gene deletion in tubular epithelial cells. Deletion of key mesenchymal proteins, Snail1 or Snail1/TWIST, in tubular epithelial cells resulted in reduced levels of interstitial fibrosis across three different disease models (UUO, folic acid nephropathy, and nephrotoxic serum nephritis) (Grande et al., 2015; Lovisa et al., 2015). A subset of tubular cells were shown to lose their epithelial markers and acquired mesenchymal markers, such as vimentin, without leaving the tubules, thereby undergoing a process of de-differentiation, rather than a complete transition into fibroblasts (Grande et al., 2015; Lovisa et al., 2015). Of note, inducible over-expression of c-Jun, and thereby over-activation of AP-1, in the tubular compartment is sufficient to induce tubular atrophy, severe tubulointerstitial fibrosis and renal failure (Wernig et al., 2017).

### JNK in tubular cell and fibroblast activation

Tubular epithelial cells are a major site of JNK activation in kidney disease and systemic JNK blockade demonstrates a pathologic role for this pathway in models of tubulointerstitial damage and fibrosis. However, since JNK signaling can be activated in many cell types, including endothelial cells and infiltrating macrophages (Flanc et al., 2007; de Borst et al., 2009), systemic blockade does not provide clear delineation of JNK function in tubular epithelial cells in disease. In the absence of studies using conditional gene deletion, this issue has been addressed using cultured tubular epithelial cells.

Tubular epithelial cells are a major source of chemokines (e.g., CCL2) and pro-fibrotic factors (e.g., TGF-β1, PDGF-B) in tubulointerstitial fibrosis (Chow et al., 2006; Ma et al., 2007b; de Borst et al., 2009), and this is suppressed by systemic JNK blockade (Ma et al., 2007a; de Borst et al., 2009). Consistent with these findings, studies in cultured tubular epithelial cells have shown that IL-1β and TGF-β1 induced MCP-1 expression (de Borst et al., 2009), and IL-1 and TNF-α induced IL-6 production (de Haij et al., 2005), operate in a JNK-dependent fashion. Similarly, tubular cell secretion and activation of TGF-β1 in response angiotensin II and aristolochic acid stimulation operates via JNK signaling (Naito et al., 2004; Ma et al., 2007a; Rui et al., 2012). Finally, the transition of tubular epithelial cells toward a mesenchymal phenotype in response to TGF-β1, alpha 2-antiplasmin, or aristolochic acid operates via JNK signaling (Mariasegaram et al., 2010; Zhou et al., 2010; Kanno et al., 2014).

While JNK activation in tubular cells and infiltrating macrophages can promote fibroblast activation and collagen production, it is less clear whether JNK signaling is important in the fibroblast itself. Studies of cultured fibroblasts have identified a role for JNK signaling in proliferation induced by PDGF, TGF-β1, and aldosterone (Khalil et al., 2005; Huang et al., 2012). However, the role of JNK in cell proliferation is highly context dependent. For example, PDGF-B induced proliferation is JNK dependent in pulmonary artery fibroblasts isolated from animals exposed to hypoxia but not in fibroblasts from control animals (Panzhinskiy et al., 2012), and PDGF-induced proliferation of pericytes was not affected by JNK inhibition (Ren et al., 2013).

In summary, JNK activation in tubular epithelial cells can promote cell death following severe injury or promote progressive tubulointerstitial inflammation and fibrosis in response to a less severe chronic insult. This chronic pathologic process is also promoted by JNK activation in interstitial macrophages, fibroblasts, and endothelial cells.

## JNK and TGF-β1/SMAD signaling

JNK interacts with a number of pathways in the fibrotic process of which the best characterized interaction is with the TGF-β/SMAD pathway. The TGF-β/SMAD signaling pathway plays a central role in the development of renal fibrosis (Meng et al., 2016). This is regulated at many levels including; TGF-β production and secretion, activation of latent TGF-β, and the activity of the SMAD2/3/4 complex to promote transcription of pro-fibrotic molecules (Meng et al., 2016). JNK signaling can enhance pro-fibrotic TGF-β/SMAD signaling through at least three distinct mechanisms. First, JNK activation induced by factors such as angiotensin II, IL-1, TNF-α, and aristolochic acid can increase TGF-β production through activation of AP-1 which binds to distinct sites in the *Tgfb* gene promoter to increase transcription (Kim et al., 1990; Naito et al., 2004; Lee et al., 2006; Rui et al., 2012). In addition, TGF-β induces a positive feedback loop to continue MAPK stimulation via TAK1, another upstream p38/JNK activator (Yamaguchi et al., 1995). Second, JNK signaling can enhance tubular production of thrombospondin-1 which, in turn, induces activation of the latent form of TGF-β1 (Naito et al., 2004). Third, JNK can directly phosphorylate residues in the linker regions of SMAD3 which enhances SMAD3 transcriptional activity (Velden et al., 2011). In addition, a direct interaction between JNK and SMAD3 has been shown in the UUO model of renal fibrosis (Sun et al., 2013). Immunoprecipitation studies showed increased levels of JNK activation as well as increased phosphorylation of SMAD3 at its linker region are very early events following UUO surgery. This interaction is also important in stimulating fibroblast proliferation and collagen production (Sun et al., 2013). However, the JNK pathway can also act to limit TGF-β/SMAD signaling. In contrast to the other SMAD proteins, SMAD7 is a negative feedback inhibitor. It competes with the SMAD2/3 complex for binding to the TGF receptor, thereby decreasing TGF-β signaling (Yan and Chen, 2011). Mice deficient in SMAD7 have increased susceptibility to renal fibrosis, whereas those with overexpression of SMAD7 have attenuated fibrosis (Chen et al., 2011). TGF–β induced up-regulation of inhibitory SMAD7 in enhanced by JNK signaling (Uchida et al., 2001).

## Targetting kinases upstream of JNK in renal fibrosis

Targeting JNK directly using small molecule compounds has identified a pro-inflammatory and pro-fibrotic role for JNK in most, but not all, types of experimental kidney disease. However, given that JNK has a clear function in normal physiology, as shown by fetal lethality of combined *Jnk1/Jnk2* gene deletion (Yang et al., 1997), it may be desirable to target only JNK signaling induced by specific stimuli. In theory, this can be done by blocking individual enzymes in the upstream MAP2K or MAP3K families, although this may also result in inhibiting other signaling pathways. One example is transforming growth factor β-activated kinase 1 (TAK1/MAP3K7), a member of the MAP3K family (Wang et al., 2001). As the name implies, TAK1 can be activated by the pro-fibrotic factor TGF-β1, although the LPS receptor (TLR4) and the IL-1 and TNF-α receptors are more potent activators of this enzyme. TAK1 signaling leads to activation of JNK, p38 MAPK, and NF-kB pathways (Wang et al., 2001; Dai et al., 2012). As *Map3k7* gene deletion is embryonic lethal, conditional global gene deletion was used to investigate this pathway in the UUO model (Ma et al., 2011). Short term *Map3k7* deletion significantly reduced renal fibrosis and inflammation in the obstructed kidney. This was associated with inhibition of JNK, p38 and NF-kB activation and so a specific role for TAK1/JNK signaling could not be distinguished (Ma et al., 2011). While the TAK1 inhibitor 5Z-7-oxozeaenol has been shown to inhibit the development of diabetic nephropathy (Xu et al., 2016), this finding is difficult to interpret as this compound inhibits a wide range of kinases including PDGFR-α, VEGR-R3, Flt3, VEGF-R1, and MEK1 (Jogireddy et al., 2009).

A MAP3K family member, apoptosis signal-regulating kinase 1 (ASK1/MAP3K5) activates only JNK and p38 pathways (Tobiume et al., 2001). In addition, *Map3k5* gene deficient mice are viable and healthy (Gerits et al., 2007) indicating that ASK1 is not essential in physiologic JNK and p38 signaling. *Map3k5* deficient mice exhibit substantial protection from both renal fibrosis and tubular cell apoptosis in the UUO model (Ma et al., 2014). This protective effect was associated with a partial inhibition of JNK activation and a complete inhibition of p38 MAPK activation. Since selective p38 MAPK inhibitors can also suppress interstitial fibrosis in the UUO model (Stambe et al., 2004), this finding does not distinguish between the individual contributions of ASK1/JNK vs. ASK1/p38 signaling. Similarly, the ability of an ASK1 inhibitor to suppress diabetic glomerulosclerosis was associated with partial JNK inhibition and complete inhibition of p38 MAPK signaling (Tesch et al., 2015).

## Future studies and therapeutic potential

Most studies in models of renal fibrosis have relied upon pan-JNK inhibitor drugs. However, many questions remain unanswered. In particular, we have little understanding of the relative contribution of JNK1 vs. JNK2 in different types of renal injury. Also, the use of systemic inhibitors does not provide detailed information regarding the function of JNK activation in different cells types following renal injury. Conditional gene deletion will be important to further our understanding of how JNK signaling promotes different aspects of kidney disease. This will also be important for determining whether we should try and deliver JNK inhibitors to specific cell types, such as tubular epithelial cells, rather than systemic administration. In addition, we need to understand the upstream components in JNK activation in different cell types as this could provide alternative therapeutic targets.

Mixed progress has been made in clinical trials of JNK inhibitors. Following the demonstration that JNK blockade could suppress renal fibrosis (Ma et al., 2007a), JNK inhibitors have been shown to alleviate fibrosis in lung disease (van der Velden et al., 2016) and liver disease (Gautheron et al., 2014). However, a phase 2 trial of the JNK inhibitor CC-930 in idiopathic pulmonary fibrosis was halted due to acute liver toxicity, although reductions in markers of lung fibrosis were noted (van der Velden et al., 2016). This has resulted in development of another JNK inhibitor, CC90001, which is in a phase I trial in patients with idiopathic pulmonary fibrosis (NCT02510937). The JIP1 inhibitor Brimapitide (also known as AM-111 or XG-102) (Vivès et al., 1997) is currently in clinical trials to reduce intraocular inflammation post-cataract surgery (Chiquet et al., 2017), and to prevent acute sensorineural hearing loss (Suckfuell et al., 2014). However, this peptide-based JNK inhibitor has yet to be evaluated in models of renal fibrosis. One alternative strategy to target JNK/p38 MAPK signaling is blockade of the upstream kinase, ASK1, which is currently in phase 2 clinical trials in patients with stage 3/4 diabetic kidney disease (NCT02177786) and in patients with non-alcoholic steatohepatitis associated liver fibrosis (NCT02466516).

In conclusion, pharmacological blockade of JNK signaling has clear potential for the treatment of acute and chronic kidney diseases. However, whether this is best achieved by systemic JNK inhibition, cell-type specific delivery of JNK inhibitors, use of isoform-specific JNK inhibition, or via targeting an upstream component of the JNK pathway has yet to be determined.

## Author contributions

All authors determined the scope and structure of the review. KG led the drafting of the manuscript. All authors critically revised the manuscript and approved the final version.

### Conflict of interest statement

DN-P has received research funding and/or compounds from Celgene for studies of JNK inhibitors (CC-401 and CC-930) in kidney disease and research funding and/or compounds from Gilead for studies of an ASK1 inhibitor (GS-444217) in kidney disease. These funders were not involved in the scope, analysis, interpretation, conclusions or writing of this review article. The other authors declare that the research was conducted in the absence of any commercial or financial relationships that could be construed as a potential conflict of interest.
